# Trust, but verify: the importance of additional imaging after BCVI screening

**DOI:** 10.1186/s13017-026-00694-y

**Published:** 2026-04-05

**Authors:** Jessica L. Masch, John R. Austin, Paul Jaffray, Louis Perkins, Jarrett E. Santorelli, Jessica L. Weaver

**Affiliations:** 1 Department of Surgery, Division of Trauma, Surgical Critical Care, Burns, and Acute Care Surgery, University of California San Diego Health, 200 West Arbor Drive, #8896, San Diego, CA 92103-8896 USA; 2 Department of Radiology, University of California San Diego Health, San Diego, CA USA

**Keywords:** BCVI, Screening, Follow-up imaging, Management

## Abstract

**Background:**

Clinical practice guidelines recommend screening for blunt cerebrovascular injury (BCVI) based on the Denver Criteria. BCVI is typically treated with antithrombotic therapy, which may be high-risk in patients with concomitant brain or spine injuries. At our center, we often perform additional imaging in high-risk patients after positive initial screening for BCVI. Our study investigates how frequently follow-up imaging changes diagnosis and management of patients screening positive for BCVI.

**Methods:**

A cross-sectional study of all trauma patients admitted to a level 1 trauma center with cervical spine or facial fractures between May 2019 and December 2022 was performed. Chart review was conducted to identify all patients who had a positive screening study for BCVI. Individual charts were reviewed for BCVI screening and additional imaging test modality and results, as well as timing and specifics of treatment recommendations.

**Results:**

A total of 2668 patients met inclusion criteria. 1407 of these patients received BCVI screening, 11 with Magnetic Resonance Angiography (MRA) and 1396 with Computed Tomography Angiography (CTA). Of those screened, 254 (9.5%) had with positive or equivocal findings, of which 197 (77.6%) patients received a second study, including 2 patients who received multiple studies. Additional imaging studies resulted in a change in the diagnosis in 96 patients (48.7%), including 105 studies (53.3%) that found no injury or chronic findings. The results of additional studies changed patient management in in 64 (32.5%) cases.

**Conclusions:**

In our study, follow-up BCVI imaging frequently identified discordant findings in a third of screening tests. This suggests that starting antiplatelet or anticoagulation therapy based on screening studies alone could result in overtreatment in a high-risk patient population.

## Background

 Blunt cerebrovascular injury (BCVI) occurs in only 0.2–2.7% of blunt trauma, but it is associated with significant morbidity and mortality [1]. The incidence of stroke after BCVI is approximately 20% [2]. Furthermore, the majority of neurologic insults secondary to BCVI occur within the first 72 h following injury [3]. Because of the significant consequences of this injury, rapid and accurate diagnosis is crucial.

It is critical to identify BCVI rapidly to prevent stroke. Prompt initiation of treatment decreases the incidence of ischemic insult. Left untreated, the incidence of stroke is 30–40% in patients with carotid injuries and 10–15% in vertebral injuries [4–8]. Antiplatelet or anticoagulant therapy is the mainstay of care [3], and with treatment the occurrence of stroke is decreased significantly to 0.5-5% [5, 9]. Thus, the accurate and timely identification of BCVI is paramount in trauma patients in order to decrease the risk of major neurologic insults.

Choosing appropriate patients to screen for BCVI has long been a subject of investigation. Historically, BCVI was rarely discovered until after the development of neurologic symptoms. In 1999, Biffl et al.. first noted the relatively large risk to patients suffering high-energy blunt trauma with certain injury patterns, including certain facial fractures, cervical spine fractures, Glasgow Coma Scale (GCS) < 6, and diffuse axonal injury [10]. This initiated widespread efforts to create effective screening tools to capture patients with BCVI. The Denver Criteria have been validated as a screening tool to identify patients who should undergo BCVI screening at the time of admission [1]. The Memphis Criteria and Eastern Association for Trauma (EAST) Criteria are also validated, and the majority of trauma centers utilize one of these guidelines in their protocols [11, 12] to identify patients that should receive screening for BCVI.

Modalities used to diagnose BCVI have changed over time. Digital subtraction angiography (DSA), previously the standard of care for screening and still the gold standard for diagnosis, has largely been replaced by computed tomography angiography (CTA), which is faster, less costly, less cumbersome, and carries a smaller risk of complications [13]. Adoption of CTA as the standard screening test for BCVI protocols has resulted in an 8-fold increase in the detection of BCVI and has decreased the time to diagnosis and initiation in treatment [14, 15]. Despite its widespread use, there are limitations in CTA’s ability to accurately diagnose or exclude BCVI, including a significant false positive rate, particularly for lower grade injuries [16, 17]. Due to this, some institutions routinely perform additional testing with either DSA or magnetic resonance imaging (MRI)/magnetic resonance angiography (MRA) after a positive CTA. More recently, certain modalities of MRA have been found to be reliable for specifically evaluating vessel walls for potential injuries, including double inversion recovery black blood imaging (DIR-BBI), vessel wall imaging (VWI), magnetization prepared rapid acquisition echo (MPRAGE), three-dimensional simultaneous non-contrast angiography and intraplaque hemorrhage (3D-SNAP), and diffusion weighted imaging (DWI). While earlier studies found no significant differences in detection rates between MRI and CTA [18], the emergence of newer modalities is now challenging this conclusion [19].

The mainstay of treatment of BCVI is antiplatelet or anticoagulation [2]. In patients with recent major trauma, concomitant injuries including intraabdominal trauma or traumatic brain injuries can make the decision on timing of initiating antithrombotic or anticoagulant therapy more complex. This presents a potential harm to overtreating BCVI seen on screening tests, as initiating these medications could worsen hemorrhage in the setting of trauma.

The purpose of this study is to investigate how often patients screened for CTA at our institution undergo follow-up imaging, as well as how frequently additional imaging changes the diagnosis or management of patients at risk for BCVI. We hypothesized that follow-up imaging rarely changed the diagnosis or management of patients with BCVI seen on screening CTA.

## Methods

Institutional review board approval was obtained and a waiver of informed consent granted for this study. A retrospective cross-sectional cohort study was performed using the trauma registry of a level 1 academic and urban trauma center to identify all patients with facial or cervical spine fractures between May 2019 and December 2022. Facial fractures and cervical fractures were chosen as the criteria to build the cross-sectional cohort, as we wanted to sample a population of patients whose injury pattern reflected a high likelihood of receiving BCVI screening under our institution’s protocol. Our institution utilizes a modified Denver criteria. Patients at our institution receive screening CTA in the event of overt signs of BCVI including potential arterial hemorrhage from the nose, neck, or mouth, a carotid bruit, expanding cervical hematoma, focal neurologic deficit, evidence of stroke on imaging, or a focal neurologic deficit. Additional mandatory screening criteria include high energy mechanisms with cervical spine fractures or ligamentous injuries, displaced mid-face fractures, mandible fractures, basilar skull fractures, temporal bone fractures, seatbelt signs over the neck, or a strangulation injury. Screening is also considered on a conditional basis at our institution for hyperextension mechanisms, traumatic brain injuries with thoracic injuries, scalp degloving injuries, thoracic vascular injuries, blunt polytrauma, or blunt cardiac rupture. These criteria are adapted from the 2020 EAST guidelines [20].

Of the registry data used for the study, demographics, length of stay, and outcomes data were abstracted. All charts were then manually reviewed for the presence or absence of a BCVI screening study. Further chart review was performed on those patients with a positive screening test to abstract imaging findings, vessel involved in injury, treatment recommendations based on screening imaging, additional testing type and results, timing between tests, and final treatment plans. At our institution, a positive BCVI screening test prompts neurosurgical consultation, which frequently leads to recommendations for additional imaging and management. Given the retrospective design of this study, these clinical decisions influenced both which patients underwent further imaging and the type of imaging obtained. The vast majority of patients at our institution undergo MRI as the second study. The timing of a second test after screening exam is variable and depends on patient stability and resource availability (e.g. MRI availability). Descriptive statistics were used to describe the frequency of screening findings, second studies, and changes in diagnosis or treatment. Because such a low number of the patients in our cohort had a reported Denver grade of injury, the data was also re-analyzed by an experienced trauma radiologist at our institution and grade of injury based on their evaluation of the screening CTA was also collected. Patients with multiple injuries identified on screening test were assigned the most severe injury grading given for the purposes of this study. Diagnosis changes were defined as injuries identified on screening studies that were not seen on subsequent imaging, or discrepancies in injuries reported between studies. Changes in management were defined as the initiation or discontinuation of medications in response to imaging findings, or the performance of an intervention based on study results.

Our primary outcome was the frequency with which secondary MRA changed the diagnosis and management of patients screening positive or equivocally for BCVI on screening CTA. Multivariable logistic regression analysis for diagnosis change for any grade of injury was also performed accounting for age, sex, race, injury severity score (ISS), highest abbreviated injury scale 1990 revision (AIS-90) score for the head/neck, location of injury, and time between studies. Multivariable logistic regression analysis of the patients with injuries identified by our institution’s radiologist to adjust for patient age, sex, ISS, AIS-90 for head/neck, vertebral versus carotid injury, and the time between first and second test in days.

Figure 1.

**Fig. 1:**
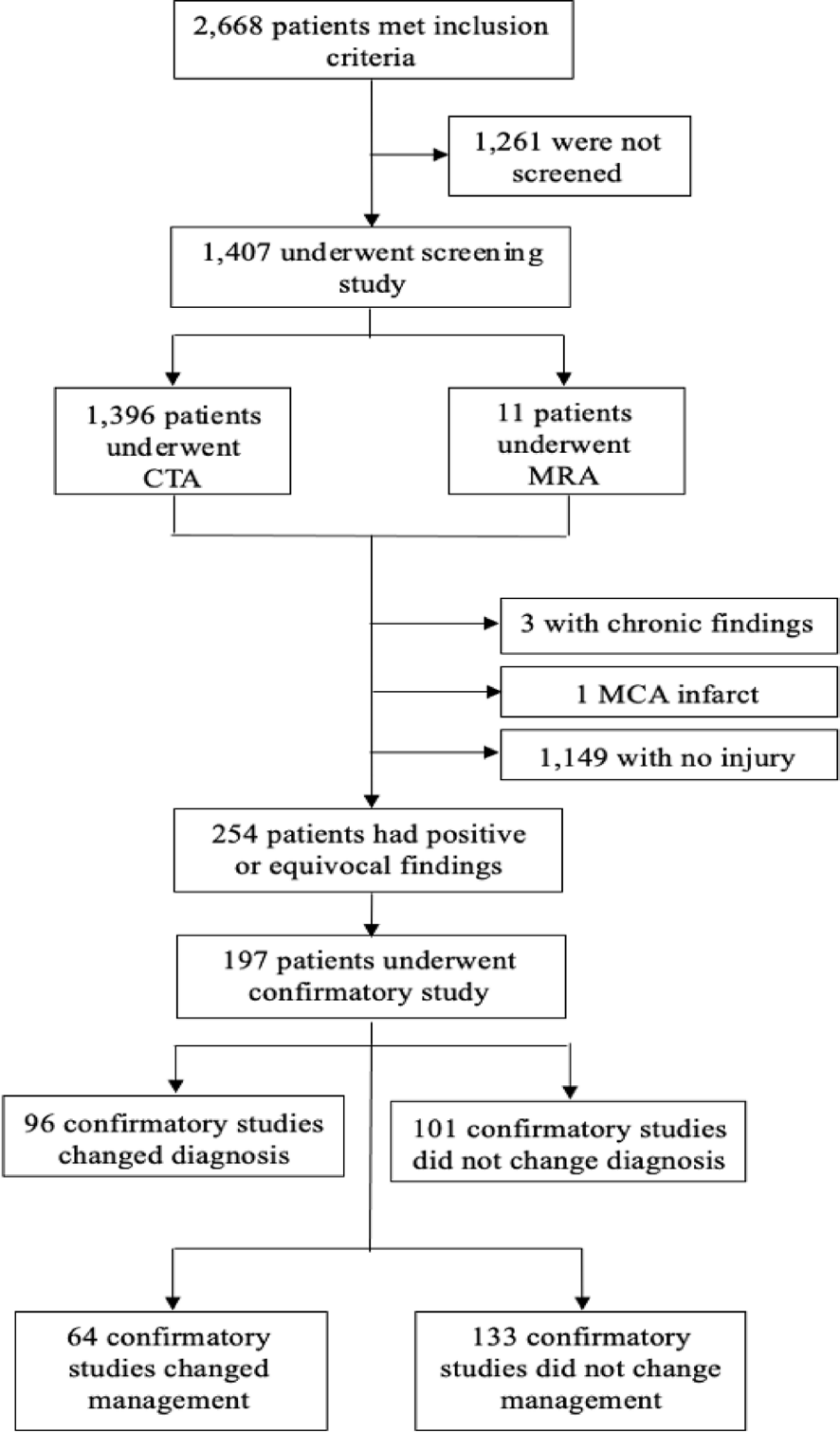
Flow chart demonstrating patients included in study

## Results

There were 2668 patients identified with facial or cervical spine fractures, of which 1407 (47.3%) underwent a BCVI screening study: 1396 with CTA and 11 with MRA. In total, 254 (9.5%) patients were positive for BCVI (Fig. 1). The cohort that screened positive was 68.5% male, 44.1% white, and had a mean age of 50.1 years. Concomitant TBI was present in 37% of patients, as shown in Table 1.

**Table 1:** Demographic information

	Patients that underwent BCVI screening (*n* = 1407)	Patients with positive BCVI screening study (*n* = 254)
Mean age (years)	48.1	50.1
Gender Male Female	1046 (74.3%) 361 (25.7%)	174 (68.5%) 80 (31.5%)
Race White Hispanic Black Asian Other/Not reported	617 (43.9%) 477 (33.9%) 106 (7.5%) 29 (2.1%) 178 (12.6%)	112 (44.1%) 89 (35.0%) 21 (8.3%) 7 (2.8%) 25 (9.8%)
Median GCS	15	15
Mean BMI	26.8	27.1
Concomitant TBI	383 (27.2%)	94 (37%)
Screening CTA	1396 (99.2%)	251 (98.8%)
Screening MRA	11 (0.8%)	3 (0.2%)

GCS, Glasgow Coma Score; TBI, traumatic brain injury; BMI, body mass index; CTA, computed tomography angiography; MRA, magnetic resonance angiography

**Table 2:** Injury grade and location on patients screening positive on CTA as read by expert radiologist

Injury characteristic	Number of patients (*n* = 78)	Diagnosis change based on second study	Management change based on second study
Injury grade			
Grade I	8 (10.5%)	4 (50%)	1 (12.5%)
Grade II	27 (34.6%)	7 (25.9%)	9 (33.3%)
Grade III	13 (16.7%)	0 (0.0%)	7 (53.8%)
Grade IV	28 (36.8%)	3 (10.7%)	13 (46.4%)
Grade V	2 (2.6%)	0 (0.0%)	1 (50.0%)
Injury location			
Vertebral artery	45 (57.6%)	8 (17.8%)	17 (37.8%)
Carotid artery	27 (34.6%)	4 (14.8%)	9 (33.3%)
Involving both carotid and vertebral arteries	6 (7.7%)	2 (33.3%)	3 (50.0%)

Table 2 shows the number of patients with each injury grade and vessel affected, and the frequency with which these subpopulations had their diagnosis or management changed by the second study

Of the 1396 patients undergoing screening CTA, 251 patients were positive for BCVI, of which 195 (77.7%) underwent a second study: 178 patients (91.3%) with MRA, 1 patient (0.005%) with ultrasound, 8 patients (0.04%) with DSA, 3 patients (0.02%) with repeat CTA, and 5 patients (0.03%) with more than one additional study (Table 1). Among the 178 patients who underwent MRA as a second test, 74 (41.6%) patients did not have BCVI on MRA. Of note, 12 (6.7%) of these patients had a non-diagnostic result, or the study had to be aborted prior to completion, usually due to patient agitation or discomfort. Additionally, 86 of the MRAs (48.3%) resulted in a change in diagnosis compared to the screening study, and 56 (31.5%) resulted in a change in the management of the patient. The second study was performed in the first 48 h in 166 (85.1%) of cases. The second study was performed between 48 and 96 h in 16 (8.2%) of patients, and after 96 h in 11 (5.6%) of patients.

Of the 251 patients who screened positive on their admission CTA, 114 patients (45.4%) were treated with anticoagulation or antiplatelet medications based on screening test result alone. Another 8 patients (0.03%) underwent a procedure, usually DSA (sometimes with stent placement or embolization). 129 patients (51.4%) were not started on any treatment based on their screening test result alone, and only secondary imaging was ordered in response to a positive or equivocal first test.

Of the 11 patients screened with MRA, 8 patients had no injury. 7 of these patients did not undergo any additional testing, and 1 underwent a CTA as a second test. Of those with injuries found on MRA, 1 patient did not undergo additional testing, 1 underwent ultrasound as a second test, and 1 underwent a DSA as a second test. Of the patients undergoing second studies after MRA, the diagnosis was changed in 2 patients (66.6%). The treatment plan was not changed in any patients. In total, 96 (48.7%) of the 197 s studies resulted in a change in diagnosis, and the treatment plan was changed in 64 (32.5%) instances. The initiation and changes in therapy based on first and second studies are outlined in Table 3. The frequency of different screening and second test combinations and their likelihood of changing diagnosis and management are shown in Table 4.

Of the 195 patients who had a positive screening CTA result, the grade was not reported in the radiology report in 179 (91.8%) of cases, which presented challenges in drawing conclusions related to lower versus higher grade injury from our dataset. For this reason, we asked an expert radiologist with robust trauma experience to review the 195 positive or equivocal screening CTAs to assign them a Denver grade. On this analysis, 99 (50.8%) of the screening CTAs associated with follow-up imaging studies were overread as not having an injury. 52 (52.5%) of these had a read of a “possible” injury on the initial screening study, and while grading data was very sparsely available for the initial screening interpretations, of those available they notably included only grade I and II injuries. On the follow-up MRI for the patients overread by the expert radiologist to have no injury, 66 (66.7%) of these patients had no injury identified or chronic findings, 7 (7.1%) were non-diagnostic, and 25 (25.3%) demonstrated a persistent injury. This discrepancy between our expert radiologist’s reads and MRA findings may be due to increased sensitivity of MRA in identifying low-grade intimal injuries or subtle wall irregularities not detectable by CTA, or due to the possible evolution of injuries due to the temporal separation between the studies. Imaging could not be reviewed for 18 (18.2%) studies, as these studies were performed at outside facilities and the images were not available for review in our system. Of the 78 screening CTAs that were positive and graded by our expert radiologist, 8 (10.5%) were grade I, 27 (34.6%) were a grade II, 13 (16.7%) were a grade III, 28 (36.8%) were grade IV, and 2 (2.6%) were grade V. 44 (57.9%) involved the vertebral artery only, 27 injuries (34.6%) involved the carotid only, and 6 (7.7%) injuries involved both the carotid and vertebral vessels, as shown in Table 2. Of the 76 patients read by our institution’s radiologist to have an injury on screening CTA who underwent secondary imaging, the diagnosis was changed by the secondary imaging in 14 (18%) cases, and the treatment was changed based on secondary test results in 29 (39.2%) cases.

A multivariable regression analysis was performed for the patients read to have an injury by the expert radiologist to assess for other factors associated with discordance between the initial screening CTA and the second test. Patient age, female sex, ISS, AIS-90 score head and neck, vertebral injury, carotid injury, and time in days between studies were assessed. None of these reached statistical significance in the regression analysis. This is summarized in Table 5. Odds ratios were not estimable by individual injury grade as these groups were too small for analysis.

**Table 3:** Management based on imaging

	After positive screening study (*n* = 254)	After second imaging study (*n* = 197)	After second study in cohort graded by expert radiologist (*n* = 76)
Therapy initiated			
Antiplatelet initiation DSA With intervention Surgical intervention Therapeutic anticoagulation	112 (44%) 9 (3.5%) 3 (1.2%) 0 (0.0%) 4 (1.2%)	46 (23.4%) 2 (1.0%) 2 (1%) 1 (0.5%) 2 (1.0%)	25 (32.9%) 2 (2.6%) 2 (2.63%) 1 (1.3%) 2 (2.63%)
Treatment stopped		12 (6.1%)	0 (0.0%)

ASA, acetylsalicylic acid (Aspirin); DSA, digital subtraction angiography

**Table 4:** Screening and confirmatory study combinations with resulting changes in diagnosis and treatment based on original radiology reads (no grade)

Screening study	Second study	*N*	Diagnosis changed	Treatment changed
CTA	MRA	178	86 (48.3%)	56 (31.5%)
CTA	Angiography	8	4 (50.0%)	3 (37.5%)
CTA	Repeat CTA	4	1 (25.0%)	1 (25.0%)
CTA	Ultrasound	1	1 (100%)	0 (0%)
CTA	Multiple studies	5	2 (0.4%)	4 (80.0%)
MRA	Ultrasound	1	1 (100%)	0 (0%)
MRA	Angiography	1	1 (100%)	0 (0%)
MRA	CTA	1	0 (0%)	0 (0%)

CTA, computed tomography angiography; MRA, magnetic resonance angiography; Angiography, digital subtraction angiography

**Table 5:** Multivariable logistic regression for diagnosis change

Predictor	Odds ratio (95% CI)	*p*-value
Age (years)	1.00 (0.95–1.04)	0.878
Female sex	2.19 (0.53–9.35)	0.275
ISS	1.03 (0.95–1.13)	0.454
AIS-90 value for head/neck	0.52 (0.13–1.82)	0.321
Vertebral injury on CTA	3.54 (0.25–52.31)	0.337
Carotid injury on CTA	4.06 (0.27–71.84)	0.307
Days between screening and second study	1.62 (0.98–2.85)	0.071

Odds ratios are adjusted for all covariates shown. Reference categories: Sex, Male; Vertebral Injury on CTA, No; Carotid Injury CTA, No

## Discussion

We sought to elucidate the impact of additional testing at our institution after positive BCVI screening and hypothesized that second tests were not likely to influence management. In fact, we found that in 42% of cases, findings on a screening CTA were not re-demonstrated on MRA, which resulted in a change in the diagnosis and approach to treatment in 48% and 32% of patients, respectively.

The limitations of CTA demonstrated in this study have been similarly reported in the literature. The advent of CTA revolutionized the screening process for BCVI and greatly increased the early detection rate of this injury [9, 19, 21, 22], but it remains an imperfect test. As CT scans have evolved to capture thinner slices with enhanced detail, their capacity to detect BCVI has significantly improved [23]. Nonetheless, the accuracy of CTA as a diagnostic study has been called into question by multiple studies due to its variable sensitivity, even with the introduction of higher multidetector sequences [15, 19, 21, 24], as well as for its relatively low positive predictive value [17]. Specifically, CTA seemingly overcalls grade I injuries, and a previous study confirming CTA results with DSA found CTA to be incorrect 61.5% of the time, with a positive predictive value of 30% for grade I injuries [17]. Although our sample of grade I injuries was relatively small, our data shows that CTA correctly identifies grade I injuries in 3 out of 8 (37.5%) cases, while the remaining 62.5% of injuries were not redemonstrated on follow-up imaging. Despite this, CTA remains the standard recommendation for BCVI screening. The 2020 Eastern Association for the Surgery of Trauma guidelines recommend screening CTA for patients with high-risk and low-risk cervical spine injuries, as well as treatment of BCVI with antithrombotic therapy [20]. The 2024 Western Trauma Association guidelines recommend repeating screening imaging for any equivocal finding, treatment with antiplatelets for grades I-IV injury and intervention for grade V injuries, and routinely repeating CTA in 7–10 days for grade I-IV injuries to ensure no evidence of injury worsening with the option of stopping antithrombotics if the injury appears stable [25]. While our institution does not routinely repeat CTA after a positive screening test, it primarily uses another study modality to verify possible BCVI found on screening imaging which often changes management.

Our study is novel in its comparison of screening studies to second tests, particularly of CTA compared with MRA. This can be uniquely evaluated through our institution’s practices due to the standard recommendations by our neurosurgical colleagues when a screening CTA is positive, as they typically request a follow-up MRA. MRA is generally not considered a good screening tool due to its high cost and length of study time. However, it can be used to better characterize possible BCVIs seen on screening studies. MRA has been shown to reliably identify high-risk features suggestive of increased stroke risk in patients with identified BCVI, such as vessel wall abnormalities and intramural thrombus [26, 27]. Recently, literature has shown promising ability of certain modalities of MRI/MRA specifically in detection of BCVI. DIR-BBI sequencing, which suppresses the signal of arterial inflow and specifically focuses on the vessel wall, was shown to be superior at detecting intimal flaps and intramural hematomas when compared to CTA [28]. Vranic et al. demonstrated the ability of MRI-VWI to reliably detect BCVI, particularly in low-grade injuries [29]. Other modalities of MRI/MRA have been investigated including 3D-SNAP and DWI which have proved valuable in detecting BCVI [30, 31] While MRA is a more time-consuming modality, it offers the benefit of avoiding ionizing radiation, which is an important consideration in certain patient populations such as children and pregnant patients. An additional benefit of MRA is its ability, when done in conjunction with MRI brain, to detect subtle ischemic findings that could be missed or too early to be seen on CT imaging, but may increase clinical suspicion of BCVI [19]. Our study found that 77.7% of patients underwent a second study after a positive screening CTA, usually an MRA. In the MRAs, 41.6% of studies did not redemonstrate an injury. This finding is in agreement with previous studies that MRA is more sensitive in diagnosing BCVI.

The mainstay of BCVI treatment is anticoagulation or antithrombotic therapy. However, these medications are frequently contraindicated in trauma patients, who frequently have concomitant traumatic brain injuries, spine injuries, or intraabdominal solid organ injuries, and in whom the risk of bleeding is significant. Thus, delays in treatment may be attributable to other factors apart from the time required to obtain a second study alone in this patient population. More invasive therapies, such as endovascular repair or stenting, have less established indications and are frequently offered on a case-by-case basis depending on the capabilities of faculty and hospitals as well as individual risks and benefits [32]. Another consideration related to the present study is the implications of the delay in treatment that obtaining an additional study could potentially generate. While it has been shown that the greatest risk for stroke after BCVI occurs in the first 72 h after injury [3], Applebaum et al. did not find an increased stroke rate in patients who started BCVI therapy 62 h after injury in comparison to those who started therapy 11 h after injury, challenging the traditional dogma that immediate treatment is necessary to minimize stroke risks. Importantly, therapy was started prior to receiving results of second studies in 45.4% of cases in our study, which suggests that awaiting a second study did not necessarily delay treatment at our institution. Additionally, 85.1% of patients in this study received their second study within 48 h of the initial screening CTA.

While data regarding the grade of injury on screening studies was not routinely available in our initial data collection, we were able to receive grades of injuries for a smaller cohort of patients when reviewed by our expert radiologist. Unfortunately, this cohort by grade of injury was not large enough to stratify for a regression analysis. However, we did find that changes in diagnosis and management occurred in both low and high-grade injuries.

Controversy still exists regarding whether or not asymptomatic low-grade BCVI require treatment. A 2021 systematic review by Murphy et al. found that grade I injuries were more likely to heal on follow-up CTA studies, even in the absence of therapy, than higher grade injuries [32]. It is possible that vessel wall healing could have occurred between the screening and second tests in the lower-grade injuries in our study cohort, as the retrospective nature of our study precluded standardization of the amount of time between the two studies. However, when looking specifically at the cases when which diagnosis and management changed after MRA, these changes were seen across all grades of injury. In addition, the relatively short time between the two studies in most patients makes this less likely to be the primary reason for the observed differences.

Imaging for BCVI is relatively costly, particularly when patients receive multiple studies to verify their injuries. At our institution, the average hospital cost of a CTA neck is $211.85 while an MRA of the neck costs $436.73. DSA, the most expensive modality, has an average cost at our institution of $3,394.98. Cost is an important consideration, because while the average cost of a screening CTA at our institution is roughly $220 less than an MRA, the majority of patients screening positive on their CTA had a follow-up MRA. If MRA were the preferred screening modality, the cost of screening all 1407 patients screened in our study would have been roughly $615,000, over twice the $298,000 cost to screen the same population with CTA. The cost of additional MRA studies in our population was approximately $78,000, over $45,000 of which could be attributed to studies that did not change the diagnosis, and roughly $53,000 of which resulted from studies that did not change patient management. While one might consider that MRA could replace CTA as a screening modality due to the number of positive screening tests that did not redemonstrate an injury in follow-up imaging, CTA remains a more affordable option overall and, despite its limitations, ultimately saves costs. Nonetheless, the concern that CTA may not capture all BCVIs due to its variable sensitivity remains to be reconciled.

Our study proposes that follow-up studies are useful in diagnosing and making informed treatment choices for trauma patients with BCVI. Our study revealed that follow-up testing led to a change in management in approximately one-third of the cases, typically resulting in the discontinuation of an antiplatelet or anticoagulant that had been initiated. Although these medications are generally considered low-risk in the broader population, their implications are significant for patients at elevated bleeding risk following trauma, though the specific risk of long-term complications from anticoagulation or antiplatelet therapy is not captured in this study. This finding underscores the potential of follow-up studies to potentially mitigate adverse bleeding events by preventing the overtreatment of injuries identified on screening CTAs, although data on observed bleeding events was not available for our study population.

Our study has multiple strengths, including a large study population, and includes assessment of changes in diagnosis and management, not just imaging rates. Our study also has several important limitations. First, it is a single-center study, and results may not translate to all centers and all patient populations. The practice of routinely obtaining MRA as a secondary imaging test is somewhat uncommon, and does not necessarily translate to other institutions, which limits the generizability of the results. Second, our study only includes retrospective data, and therefore the timing between imaging studies was not standardized and the reasons for the clinical decisions affecting management are unable to be fully captured from the data abstracted. Third, the nature of this study in which the patients who obtained additional imaging were at the discretion of a consulting service makes it impossible to control for selection bias. Fourth, when the grade of injury was collected for our population by an expert radiologist, many of the screening CTAs read as positive by other radiologists were overread as “no injury”, limiting our cohort data with injury grade data available for analysis. However, in the patients found to have injuries by our radiologist, there was good representation of both low-grade and high-grade injuries. This highlights the importance of having an expert trauma radiologist available at level 1 centers to more accurately diagnose BCVI on screening studies in addition to high-quality CT images. Finally, we did not have any long-term outcomes data about the impact of this management or rates of bleeding events from treatment or from cerebral ischemic events related to BCVI, limiting our ability to detect a meaningful clinical benefit.

## Conclusion

In conclusion, follow-up MRA after positive screening CTA is a useful test in suspected BCVI as it frequently changed diagnosis in our study population and has the potential to prevent overtreatment of these injuries in a high-risk patient population. However, to limit costs and overutilization of resources, consideration should be given to whether the results of the study will change the patient’s clinical management.

## Data Availability

The datasets used and/or analyzed during the current study are available from the corresponding author on reasonable request.
